# Feasibility and Quit Rates of the Tobacco Status Project: A Facebook Smoking Cessation Intervention for Young Adults

**DOI:** 10.2196/jmir.5209

**Published:** 2015-12-31

**Authors:** Danielle E Ramo, Johannes Thrul, Kathryn Chavez, Kevin L Delucchi, Judith J Prochaska

**Affiliations:** ^1^ Department of Psychiatry University of California, San Francisco San Francisco, CA United States; ^2^ UCSF Helen Diller Family Comprehensive Cancer Center Tobacco Control Program University of California, San Francisco San Francisco, CA United States; ^3^ Center for Tobacco Control Research and Education University of California, San Francisco San Francisco, CA United States; ^4^ Stanford Prevention Research Center Department of Medicine Stanford University Stanford, CA United States

**Keywords:** smoking cessation, Facebook, social media, young adults

## Abstract

**Background:**

Young adult smokers are a challenging group to engage in smoking cessation interventions. With wide reach and engagement among users, Facebook offers opportunity to engage young people in socially supportive communities for quitting smoking and sustaining abstinence.

**Objective:**

We developed and tested initial efficacy, engagement, and acceptability of the Tobacco Status Project, a smoking cessation intervention for young adults delivered within Facebook.

**Methods:**

The intervention was based on the US Public Health Service Clinical Practice Guidelines and the Transtheoretical Model and enrolled participants into study-run 3-month secret Facebook groups matched on readiness to quit smoking. Cigarette smokers (N=79) aged 18-25, who used Facebook on most days, were recruited via Facebook. All participants received the intervention and were randomized to one of three monetary incentive groups tied to engagement (commenting in groups). Assessments were completed at baseline, 3-, 6-, and 12-months follow-up. Analyses examined retention, smoking outcomes over 12 months (7-day point prevalence abstinence, ≥50% reduction in cigarettes smoked, quit attempts and strategies used, readiness to quit), engagement, and satisfaction with the intervention.

**Results:**

Retention was 82% (65/79) at 6 months and 72% (57/79) at 12 months. From baseline to 12-months follow-up, there was a significant increase in the proportion prepared to quit (10/79, 13%; 36/79, 46%, *P*<.001). Over a third (28/79, 35%) reduced their cigarette consumption by 50% or greater, and 66% (52/79) made at least one 24-hour quit attempt during the study. In an intent-to-treat analysis, 13% (10/79) self-reported 7-day abstinence (6/79, 8% verified biochemically) at 12-months follow-up. In their quit attempts, 11% (9/79) used a nicotine replacement therapy approved by the Food and Drug Administration, while 18% (14/79) used an electronic nicotine delivery system to quit (eg, electronic cigarette). A majority (48/79, 61%) commented on at least one Facebook post, with more commenting among those with biochemically verified abstinence at 3 months (*P*=.036) and those randomized to receive a personal monetary incentive (*P*=.015). Over a third of participants (28/79, 35%) reported reading most or all of the Facebook posts. Highest acceptability ratings of the intervention were for post ease (57/79, 72%) and thinking about what they read (52/79, 66%); 71% (56/79) recommended the program to others. Only 5 participants attended the optional cognitive-behavioral counseling sessions, though their attendance was high (6/7 sessions overall) and the sessions were rated as easy to understand, useful, and helpful (all 90-100% agreed).

**Conclusions:**

A Facebook quit smoking intervention is attractive and feasible to deliver, and early efficacy data are encouraging. However, the 1.5-fold greater use of electronic cigarettes over nicotine replacement products for quitting is concerning.

## Introduction

Although the prevalence of cigarette smoking has declined among US adults since 1983, the smoking prevalence among young adults aged 18-25 years has remained stable, with past month cigarette use rates as high as 31% in 2013 [1]. Compared to other age groups, young adults are less likely to use behavioral or pharmacotherapy interventions for smoking cessation [2], and studies of tobacco use have reported great challenges in recruiting young adults [3,4]. Reaching the US public health goal to reduce smoking prevalence to no higher than 12% by 2050 will require novel intervention approaches, enhancement of the effectiveness of existing treatments, and maximized reach and utilization of both.

Web-based social networks may serve as a solution to the problems of reach and engagement in smoking cessation interventions for young adults. Social networks, including those developed through online social media, play a role in onset and perpetuation of smoking behavior [5,6]. Patterns of social interactions through online smoking cessation forums have characterized ways that social networks can influence smoking cessation through social support, including by offering encouragement and emotional support, stories, congratulations, “thank you” messages, giving practical advice and tips, and discussing nicotine replacement therapy [7,8]. With wide reach and engagement among users, social media tools offer phenomenal opportunity to use social interactions to engage young people in behavior change interventions and to foster socially supportive communities for quitting smoking and sustaining abstinence.

With use continuing to increase annually, Facebook remains the most widely used social media tool and the second most popular website in the United States [9] with over 156 million US users as of January 2015 [10]. With 87% of US online young adults having a Facebook account and 70% of those accessing it daily [11], there is promise to use this platform to deliver public health intervention programs to young people.

Previous evaluations using Facebook to change health risk behavior have shown feasibility as measured by participant engagement and satisfaction [12-20]. However, trials examining social media interventions have shown limited or no effects on health behavior change (eg, physical activity) [21]. As applied to smoking, the BIO smoking cessation campaign for young adults in Canada, incorporating a website, smartphone app, and Facebook features, resulted in greater 7-day and 30-day reported quit rates than referral to a smokers’ helpline at 3-month follow-up [22]. Research is needed to determine whether Facebook alone can be used as an intervention tool for smoking cessation, whether abstinence can be biochemically verified, and whether abstinence rates can be maintained over 1 year.

In a mixed-methods study, we previously [23] examined 570 young adults’ receptivity to using Facebook to quit smoking, and 31% reported they would want to get help to quit smoking using Facebook. Interest in using Facebook to quit was greater among those more motivated to quit, who had made a quit attempt in the past year, and had previously used the Internet for assistance with a quit attempt. In qualitative interviews, social support and convenience were identified as strengths of a Facebook intervention, while privacy was the main issue of concern. It was determined that an intervention delivered through Facebook should be tailored to readiness to quit smoking and should deliver evidence-based content to groups of smokers who can support one another, while maintaining privacy from larger Facebook social networks.

While retention in social media intervention studies is promising, engagement may not be high enough to promote and sustain behavior change. Given that engagement in Web interventions influences efficacy [24], and social media is fraught with some of the same engagement concerns as online interventions with large drop-offs in participation among users [25], engagement is of utmost importance in designing this type of intervention. The use of monetary incentives is successful in recruiting participants to randomized clinical trials [26] and in yielding short-term abstinence to substance use [27]; however, the use of incentives to increase engagement in social media behavior change intervention has yet to be evaluated. What type of incentive intervention works best with young adults in a Facebook intervention is an empirical question. Monetary incentives have been effective in other settings, but it is also possible that donation would be a motivator for engagement in a health behavior intervention, especially for those with high amounts of trait altruism, an intrinsic motivator [28].

Herein, we describe feasibility of the Tobacco Status Project*,* a Facebook smoking cessation intervention for young adults in the United States. The intervention was tailored to readiness to quit smoking; therefore, participants did not have to want to quit smoking to participate. Goals of this trial were to examine feasibility and initial efficacy of the intervention, including participant characteristics, retention, intervention characteristics (eg, number of groups formed, size of groups), smoking outcomes over 12 months (7-day point prevalence abstinence, ≥50% reduction in cigarettes smoked, quit attempts and strategies used, readiness to quit), engagement, and satisfaction with the intervention. To inform best practices for engagement in a larger trial, we also compared incentive conditions contingent upon daily commenting to the study. Participants could either receive a personal incentive or an incentive for a charity donation in the participant’s name. Last, we tested moderating effects by trait altruism.

## Methods

### Participants and Recruitment

Participants were aged 18-25, English literate, and had smoked ≥100 cigarettes in their lifetime, currently smoked at least 3 days per week (consistent with the definition of smoking used in the National Health Interview Survey) [29], and used Facebook at least 4 days per week. Participants had to have access to technology that could take and send an online picture for verification of tobacco abstinence with a study mailed saliva cotinine test.

Recruitment efforts included a paid Facebook ad campaign conducted between June and August 2013 with details reported previously [30]. Advertisements invited participants to a secure, confidential online survey to evaluate eligibility and, for those eligible, informed consent to participate in the intervention. Online consent questions were used to confirm understanding of study procedures. Consented participants were asked to send proof of identity either by emailing a copy of a photo ID with birth date or by “friending” the study on Facebook to determine age.

### Study Design

After completing an online baseline assessment, all participants were assigned to “secret” Facebook groups tailored to their stage of change for quitting smoking (preceontemplation, contemplation, preparation) [31,32]. As per Facebook privacy options for groups, only study administrators and group members knew the existence of “secret” groups, members of the group, or any member activity. This was distinct from a “public” group, in which anyone could join and actions were public, or a “closed” group, in which anyone could ask to join or be invited and existence/membership was not private. More information about the differences between group types on Facebook is described in Facebook’s Help Center [33]. Secret groups were chosen based on mixed-methods formative work with young adults suggesting that privacy was an important consideration that would likely affect participation in a Facebook smoking cessation intervention [23]. A stage-matched group began 2 weeks after the first participant was assigned so that no group member was kept waiting longer than 2 weeks to start the intervention; thus, group sizes varied. The intervention was delivered daily for 90 days. During the intervention, participants’ stage of change was assessed monthly and if they had advanced, they were invited to join a later stage group. As this was a feasibility evaluation, participants could join the later stage group, remain in their original group, or add a second group and participate in two. We assessed program use and acceptance at intervention end (3-month follow-up), and smoking outcomes at 3, 6, and 12 months. Participants were contacted by email and Facebook to complete online follow-up assessments and compensated for each assessment with US $20 gift cards to their choice of national retailer (eg, Amazon, Best Buy) and an additional US $20 gift card if all four assessments were completed for a total possible compensation of US $100.

### Intervention Description

#### Secret Groups

All participants received access to a secret Facebook group tailored to their stage of change: Precontemplation: “Not Ready to Quit”; Contemplation: “Thinking About Quitting”; or Preparation: “Getting Ready to Quit.” Participants were invited to participate in the secret group on Facebook. All groups received daily Facebook postings for 90 days tailored to their stage of change and consistent with US Clinical Practice Guidelines for Treating Tobacco Use and Dependence [34]. Those in the “Not Ready to Quit” groups received messages incorporating the 5Rs (relevance, risks, rewards, roadblocks, repetition) [35,36]; core Motivational Interviewing techniques of expressing empathy, rolling with resistance, supporting self-efficacy, and developing discrepancy [35,37-41]; and Transtheoretical Model strategies of increasing the pros of quitting, raising consciousness about quitting smoking, and environmental opportunities to quit smoking (eg, clean indoor air laws) [42]. “Thinking About Quitting” group posts emphasized decreasing the cons of smoking, and environmental re-evaluation (identifying negative effects of smoking on others and positive effects of change). “Getting ready to quit” posts focused on self-liberation (eg, making a commitment to quit), stimulus control (eg, removing smoking paraphernalia from the home), and counter conditioning (eg, engaging in alternative behaviors). The posts used a mix of imagery, text, and Facebook poll formats.

#### Ask the Doctor Sessions

In all groups, regardless of stage of change, weekly interactive sessions were conducted with the first author, during which participants could ask any questions related to smoking or quitting. All sessions included a post introducing the live, hour-long interactive session, and invited participants to share any questions or issues that they wanted to discuss regarding smoking or quitting. These sessions were not formally scripted to allow for participant-guided content. Responses to posts incorporated motivational interviewing in all groups, especially useful for younger smokers and those with low motivation to quit smoking [40,43] and cognitive behavioral counseling techniques for those ready to quit, recommended by US Clinical Practice Guidelines [34]. Initially, sessions were conducted at the same time each week based on participants’ reports of when they were most likely to use Facebook. As the intervention progressed, session times varied. Sessions were scheduled and run through the secret groups.

#### Cognitive Behavioral Therapy Cessation Sessions

At any time during the intervention through the 12-month follow-up, participants could opt to participate in 7 sessions of group cognitive behavioral therapy (CBT) counseling delivered through Facebook chat with the first author. Interest was assessed through secret groups, and interested participants were scheduled for an initial session with the first author. The initial session was designed to help participants set a quit date and make a specific quit plan. Participants were then assigned to a CBT group based on availability of other interested participants (not necessarily in the same secret group) and asked to attend weekly sessions through Facebook chat. At the weekly time, the counselor invited all group members to a private chat session through Facebook’s group chat feature. Content for sessions was scripted and adapted for social media delivery from a manual developed by Brown et al [40]. Sessions included text and images designed to be pasted into chat sessions by the counselor along with ad hoc responses to session-specific content. Topics in the six group sessions included (1) Preparing for Quitting; (2) Celebrating Cessation, Addressing Withdrawal & High-Risk Situations; (3) Getting Support and Asserting One’s Needs; (4) Managing Mood and Stress; (5) Living Healthy: Exercise, Food, and Substance Use; and (6) Maintaining Motivation, Graduation, and Looking Forward.

Although not directly available through the study, information was given about nicotine replacement therapy and medication for smoking cessation to all groups through posts, Ask the Doctor sessions, and CBT counseling sessions.

#### Intervention Engagement Incentives

Participants were able to interact with the intervention by “liking” or commenting on intervention posts, in Ask the Doctor sessions, or to make original posts or comment on other members’ posts. In a three-group design, we tested the utility of using incentives (none, personal, altruistic) to encourage intervention engagement. All participants were randomized to one of three incentive conditions: (1) Personal incentive: Participants were told that a US $50 gift card would be emailed if they commented on all 90 posts to their Facebook secret group by the end of the 90-day intervention period; (2) Altruistic incentive: A US $50 gift card to be donated to a charitable organization of their choice through the “justgive” website if they commented on all 90 posts; or (3) No incentive. This design was used to balance maximizing engagement (commenting on all 90 posts) with staff burden of counting all comments to Facebook group only once (at the end of the intervention period). Incentive condition was evaluated in relation to “likes” and comments, as well as 3-month abstinence. Incentive group was not hypothesized to have an effect on longer-term abstinence.

Upon completion of the intervention, data from secret groups (all likes and comments) were extracted from Facebook through the Facebook application programming interface.

### Measures

All measures were administered online using Qualtrics. Qualtrics is a secure, online, Health Insurance Portability and Accountability Act (HIPAA) compliant survey software that transmits data to and from secure, firewalled data centers using Transport Layer Security encryption, the successor to Secure Sockets Layer encryption. Secure access is available to all faculty and staff at the University of California, San Francisco.

#### Demographics

At baseline, we assessed age, gender, race/ethnicity, household income, years of education, college enrollment, and employment.

#### Smoking

A Smoking Questionnaire [44] assessed average number of cigarettes smoked per day, and days smoking per week, total cigarettes smoked in the past week, age of initiation of smoking, years of smoking, and presence of past year quit attempt (y/n). Participants’ future smoking goals were assessed using one item with 7 response options, categorized into 3 categories: (1) no goal, (2) controlled or reduced smoking, and (3) abstinence [45]. Time to first cigarette upon waking (<30 min or >30 min) measured dependence [46]. The Smoking Stages of Change Questionnaire [31] was administered at baseline, 3, 6, and 12 months.

The primary outcome was tobacco abstinence, assessed and verified at 3, 6, and 12 months as the number of cigarettes smoked in the past week (even a puff; 7-day abstinence) or use of any form of tobacco. Consensus guidelines recommend use of 7-day point prevalence abstinence in cessation induction studies with smokers unmotivated to quit, who will be quitting at different time points in a trial (ie, cessation induction trials) [47]. Those reporting “no smoking in the past 7 days” were sent by mail a semiquantitative NicAlert saliva cotinine test kit with instructions to send two photos to research staff: one showing the participant putting saliva into a single collection tube and a second of the results. Cotinine levels of 30 ng/mL or less verified abstinence.

Secondary outcomes of interest were proportion of respondents reporting 50% or greater reduction in the number of cigarettes smoked in the past 7 days between baseline and 3 months, 6 months, and 12 months, and percentage of participants reporting a 24-hour smoking quit attempt during the study as well as the number of quit attempts reported. We also assessed strategies participants used to aid cessation attempts at 3, 6, and 12 months, including cessation medication or counseling (other than our intervention), or electronic nicotine delivery systems (ENDS). Any use over the 12-month study was determined (y/n) for each strategy.

#### Engagement

Engagement was examined by quantifying the number of “likes” and comments made during the intervention period. Engagement in the CBT counseling was assessed as the number of participants who opted for CBT sessions and the mean number of sessions they attended.

#### Program Acceptance/Likability

At intervention end (3 months), an 8-item questionnaire assessed participant reaction to the Facebook group (postings and “The Dr. Is In” sessions) and each CBT session, measured on a 4-point scale from “strongly disagree” to “strongly agree.” Proportions of those reporting “agree” or “strongly agree” were computed for each item.

#### Altruism

Altruism was measured using a hypothetical version of the Dictator Game, often used in economics experiments, in which participants are given an endowment and must choose the amount to allocate to themselves and how much to another person. In our version, participants were granted US $10 and asked to consider keeping it or sending any portion of it in $1 increments, to another anonymous person [48,49]. The amount sent was regarded as a measure of other-regarding or altruism (scored between 0 [least altruistic] and 10 [most altruistic]). See Multimedia Appendix 1 for the measure used.

### Analyses

Descriptive statistics characterized the sample at baseline, evaluated the number of completed assessments at 3, 6, and 12 months, and characterized the intervention (number and size of groups, number of participants changing or leaving groups, number of participants opting to attend CBT sessions). Primary and secondary outcomes were assessed at 3, 6, and 12 months. Primary outcomes were 7-day point prevalence abstinence and biochemically verified abstinence. Secondary outcomes of interest were the proportion that made at least one 24-hour quit attempt, calculated for each time period, and for the entire 12-month follow-up period. Use of strategies to quit smoking between baseline and 12-month follow-up was summarized and predictors of strategy use were analyzed with logistic regression analyses. Wilcoxon signed rank tests compared past 7-day cigarettes smoked between baseline and each follow-up. Bowker’s test for change in a repeated categorical measures evaluated stage of change transitions between baseline and 3 months, 3 and 6 months, and 6 and 12 months (3 models). To evaluate engagement, total number of “likes” and comments to Facebook groups over 3 months were tallied. Due to skew, the non-parametric Kruskal-Wallace analysis of variance (ANOVA) evaluated comments to the Facebook groups by baseline stage of change. Mann-Whitney U tests compared comments for those with biochemical-verification of abstinence at 3 months to those non-abstinent. Kruskal-Wallace tests evaluated the effect of incentive condition (personal, altruistic, no incentive) on comments to the Facebook group, in both the full sample and only those who made at least one comment to the group to address highly skewed data. Within the altruism incentive group, Kendall’s tau test indexed trait altruism by comments. Kendall’s tau test also indexed incentive condition by 3-month reported abstinence and biochemically verified abstinence. Usability and satisfaction with the intervention were evaluated by tallying the proportion of users answering “agree” or “disagree” to each item on the usability measure.

## Results

**Figure 1:**
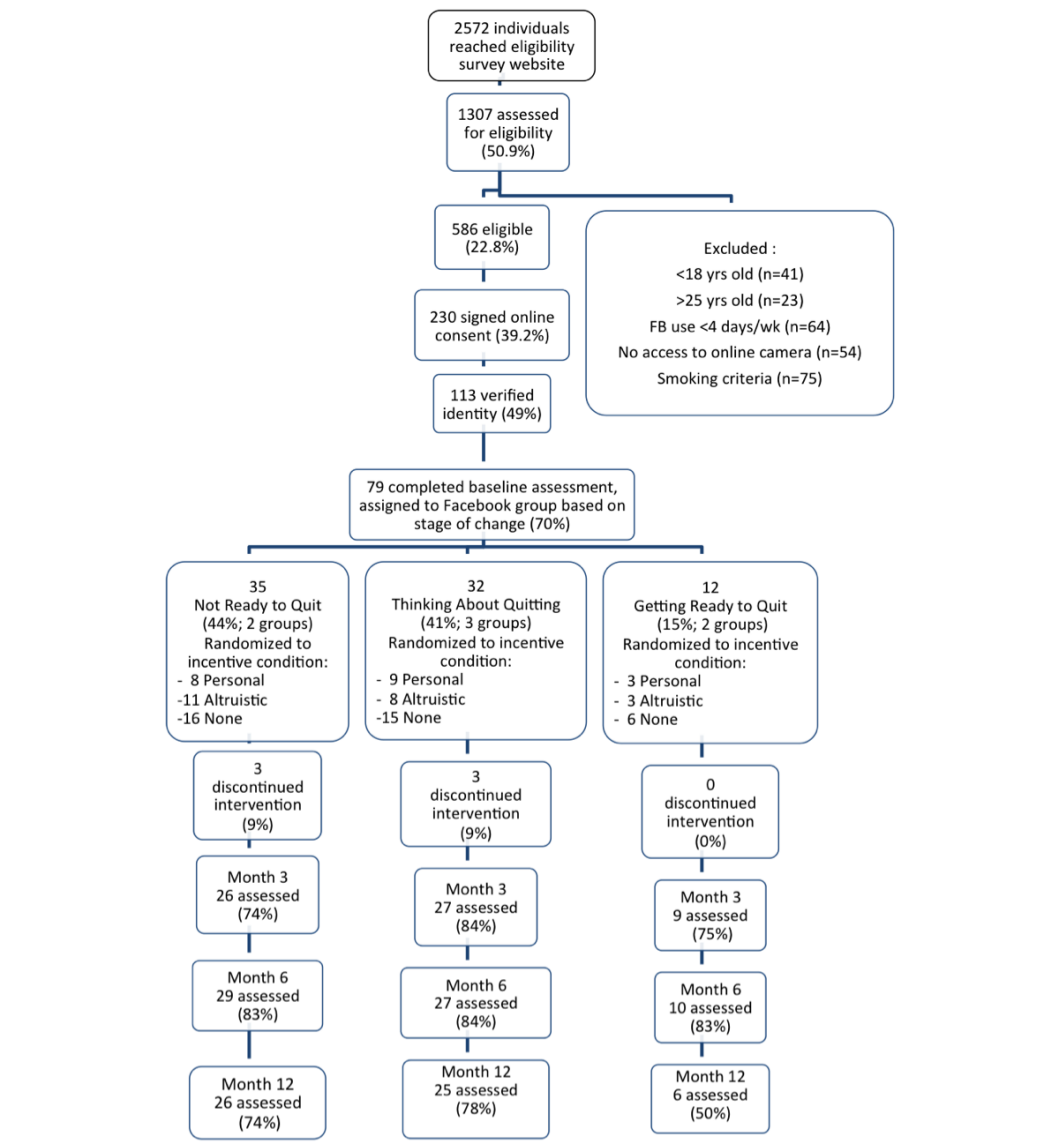
Participant flow chart through a Facebook smoking cessation intervention. Those assessed for eligibility who were not counted as “excluded” left the survey too early to determine why they were ineligible (participants were randomized to incentive groups as follows: 24% Personal; 28% Altruistic; 48% None).

Of the 586 respondents who met criteria to participate, 39.2% (230/586) signed online consent, 19.3% (113/586) verified identify online, and 13.5% (79/586) completed a baseline assessment and were assigned to a Facebook group (Figure 1).

### Baseline Demographic and Smoking Characteristics

The 79 enrolled participants who completed a baseline assessment, on average, were 20.8 years old (SD 2.1) and primarily male (63/79, 80%) and non-Hispanic white (63/79, 80%). Seventeen percent (13/79) identified as sexual minority (lesbian, gay, or bisexual). About one quarter (22/79, 28%) had a household income >US $60,000. Average years of education was 12.4 (SD 2.0); 38% (30/79) were currently enrolled in school and 56% (44/79) were currently employed; and 43% (34/79) lived with their parents. Three quarters (59/79, 75%) were daily smokers, smoking 11.5 cigarettes per day on average (SD 8.3). Only a minority (8/79, 10%) of participants reported an abstinence smoking goal, 60% (47/79) reported a reduction goal, and 30% (24/79) reported no goal. More than half (41/79, 52%) of participants smoked within 30 minutes of waking. Participants smoked their first cigarette at age 14.2 years (SD 3.4), started smoking regularly at age 16.3 (SD 2.8), and had smoked for 2.7 years (SD 0.7) on average. Over half (45/79, 57%) had made at least one smoking quit attempt in the past year.

### Retention

Follow-up completion was 76% (60/79) at 3 months, 82% (65/79) at 6 months, and 72% (57/79) at 12 months with no difference in 3-month retention by incentive condition (χ^2^_2,79_=.31, *P*=.855).

### Intervention Characteristics

Seven Facebook secret groups were created of varying sizes (two “Not Ready to Quit,” three “Thinking About Quitting,” and two “Getting Ready to Quit”; group size range 6-22). During the 90-day intervention period, 5% (4/79) of participants opted to change to a later stage group; 8% (6/79) of participants left their Facebook group completely at some point during the 3-month intervention period (3 precontemplation, 3 contemplation). Several participants (5/79, 6%) entered into CBT treatment, with one group made up of 3 participants, and 2 participants with individual treatments due to lack of other participants for groups. Participants attended 6 of 7 CBT chat sessions on average.

### Primary Smoking Outcome

**Figure 2:**
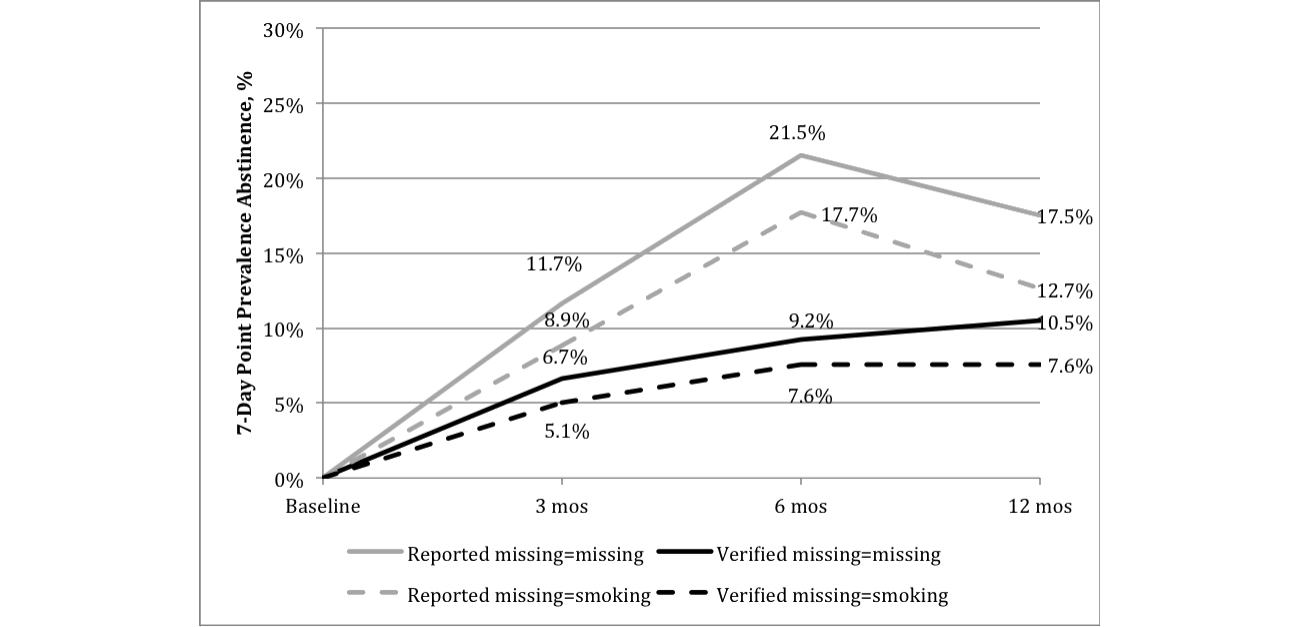
Reported and biochemically verified 7-day point prevalence abstinence by time in a Facebook smoking cessation intervention among young adults in an intent-to-treat (missing=smoking) and complete case (missing=missing) analysis (N=79). Follow-up rates are 76% at 3 months, 82% at 6 months, and 72% at 12 months. Verified abstinence includes only those who returned biochemical verification of abstinence at each follow-up assessment.

Assuming missing=missing (ie, complete case analysis), reported 7-day point prevalence abstinence was 12% (9/79), 22% (17/79), and 18% (14/79) at 3-, 6-, and 12-months follow-up (see Figure 2). Assuming those lost to follow-up were smoking (ie, missing=smoking), 7-day point prevalence abstinence was 9% (7/79), 18% (14/79), and 13% (10/79) at 3-, 6-, and 12-months follow-up. At each time point, cotinine-verified abstinence was confirmed with approximately half of those reporting abstinence.

### Secondary Smoking Outcomes

#### Quit Attempts, Cessation Aids, and Smoking Reduction

Between baseline and 12-month follow-up, 66% (52/79) of the sample reported at least one purposeful quit attempt lasting at least 24 hours: 46% (36/79) at 3 months, 40% (32/79) at 6 months, 39% (31/79) at 12 months. During the 12 months, in addition to support through the Tobacco Status Project, 11% (9/79) of participants used a nicotine replacement therapy approved by the Food and Drug Administration (5% nicotine gum, 5% nicotine patch, 1% nicotine inhaler), and 18% (14/79) reported using an electronic nicotine delivery system (ENDS) to help them quit smoking. Almost half (38/79, 48%) reported reducing the number of cigarettes smoked by 50% or greater from baseline to 3 and 6 months, and 35% (28/79) reduced at least 50% from baseline to 12 months.

#### Transitions in Stage of Change

More participants were in preparation or action at intervention-end (3-month follow-up; 32/79, 40%) than at baseline (10/79, 13%; Bowker’s X^2^=66.7, *P*<.001). At 6 months, more participants were quit or ready to quit (37/79, 47%) than at 3 months (32/79, 40%; Bowker’s X^2^=66.7, *P*<.001). Transitions from 6-12 months (36/79, 46% ready to quit) were not significant (Bowker’s X^2^=5.03, *P*=.754).

### Engagement

#### Number of Likes and Comments

Half of participants (40/79, 51%) ‘‘liked’’ at least one study-related post on the Facebook group. The median number of “likes” per person among those 40 participants was 4.0: interquartile range (IQR) 5.5; range 1-73. Almost two-thirds of participants (48/79, 61%) commented on at least one post. Two participants commented on all 90 daily postings. The median number of comments per person among those who posted or commented was 12.0 (IQR 19.5; range 1-78). Volume of commenting did not significantly differ by group, showing Precontemplation: median 0 (IQR 11); Contemplation: median 4.5 (IQR 10.8); Preparation: median 9.5 (IQR 30.5); U_2_=5.06, *P*=.080. With the full sample, comment volume was not significantly related to biochemically verified abstinence status at 3 months (U=417.5, *P*=.103). Yet among those who commented at least once (n=48), volume of commenting was significantly greater among those who quit (median 49) compared to those who did not: median 8.0 (IQR 15.0); U=115.50, *P*=.036. Comment count was not significantly related to reduction of cigarettes smoked by 50% or greater, having made a quit attempt during the study period, or readiness to quit smoking at the 12-month follow-up.

#### Incentive Effects

For the full sample (N=79), there was no significant difference among incentive conditions on number of comments made to Facebook groups: personal median 8 (IQR 19); altruistic median 2 (IQR 6); no incentive median 2.5 (IQR 11.8); χ^2^_2,79_=.035, *P*=.749. Among those who commented at least once (48/79, 61%), those in the personal incentive condition made more comments than those in the other two conditions: personal median 16 (IQR 24); altruistic median 5.5 (IQR 12.3); no incentive median 7 (IQR 12); χ^2^_2,79_=8.44, *P*=.015. There was no significant relationship between altruism and comments within the altruistic incentive group (*P*=.99). There were no difference by incentive condition on reported 3-month abstinence rates (χ^2^_2,60_=3.17, *P*=.205) or biochemically verified 3-month abstinence (χ^2^_2,60_=1.80, *P*=.406).

#### Usability and Satisfaction With Intervention

**Figure 3:**
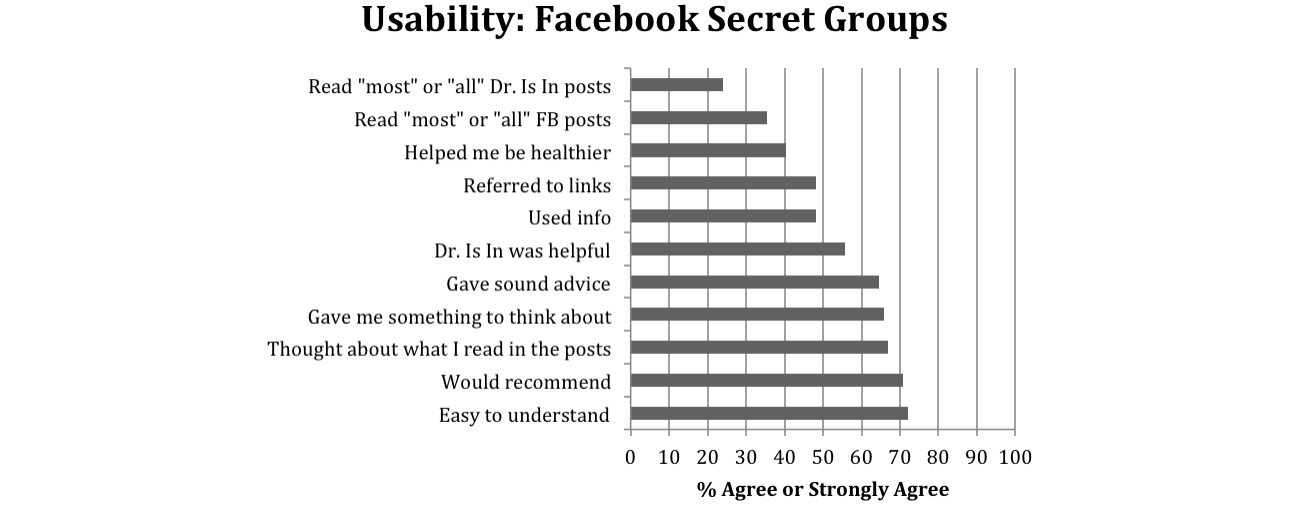
Proportion of respondents reporting they “agree” or “strongly agree” with statements about their Facebook group for a Facebook smoking cessation intervention (N=62).

**Figure 4:**
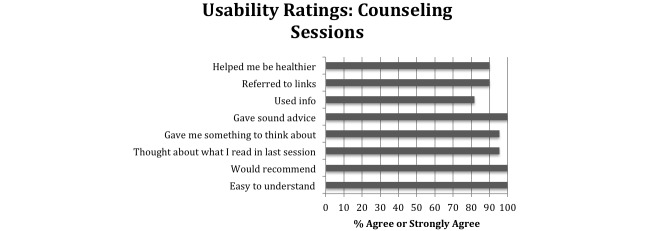
Proportion of “agree” or “strongly agree” reports about 7 cognitive-behavioral counseling sessions for a Facebook smoking cessation intervention (22 session reports).

More than a third of participants (62/79, 35%) reported reading most or all of the Facebook posts, and 24% (19/79) read most or all of “The Dr. Is In” sessions (Figure 3). Highest ratings were for post ease (57/79, 72%), thinking about what they read (52/79, 66%), and would recommend the program to others (56/79, 71%). Among the 22 CBT counseling sessions rated by 5 participants, all were rated as easy to understand, providing sound advice, and would recommend the program to others; 90% (20/22) of sessions were rated helpful; 90% (20/22) referred to material after the session; and 82% (18/22) of sessions had information that participants later used to make a behavior change (Figure 4).

## Discussion

### Principal Findings

In our pilot investigation, a novel Facebook-integrated smoking cessation intervention was found to be likable and feasible to deliver to young adults who smoke cigarettes. Early efficacy data are encouraging and support further investigation. Retention also was good, especially for a digital health intervention, though it dropped off for those in preparation at 12 months. Unknown is whether the loss to follow-up was related to quitting or relapsing. This was the smallest group, hence, the values are less stable and more subject to variability. The low sample size in this group could have led to less engagement with the intervention, subsequently leading to a lower desire to participate in follow-up surveys. However, usability data did not differ across readiness to quit groups. A trial with a sample size to support larger groups of those ready to quit is warranted.

Reported and biochemically verified abstinence increased from 3 months to 6 months and then declined slightly at 12 months, while biochemically verified abstinence was flat between 6 and 12 months. Previous trials testing the efficacy of stage-tailored interventions for smoking cessation and other health risk behaviors have found increased abstinence rates over 24 months with repeated exposure to treatment across 1 year (eg, [50]). Participants had access to Facebook groups and all associated materials over the 12-month trial; however, extending the active intervention phase (daily postings) past 3 months, or having “booster” postings over time, may enhance the efficacy of the intervention at 12 months.

Monetary incentives increased intervention uptake, but only for those who had some level of engagement (ie, commented at least once during the 3-month intervention). Further, engagement was related to abstinence only among those who commented at least once. For those motivated to engage, monetary incentives appear to be an important adjunct to a health behavior change intervention and could translate to behavior change. Monetary incentives have been identified as a main motivator for participation in online mental health interventions [51]. For smoking cessation interventions with young people, incentives have been associated with lower retention [52]. In our study, we tied a single monetary incentive (US $50) to commenting on all daily posts for 90 days. Findings suggest that a personal incentive was more effective than one in which participants provided it to others regardless of trait altruism. A more frequent incentive (eg, weekly) may further increase participation. Future studies should test various incentive schemes for engagement, balance incentives across participants and stages of change, and be powered to test the effects of groups to better determine the strategies most effective to increase engagement and for whom.

Participation in CBT counseling was relatively low, yet attendance among those who elected to receive CBT was high (6 of 7 sessions were attended on average). Conducting counseling sessions through Facebook chat was thought to provide a more personal connection. To maximize engagement, however, these sessions could be offered in a more open format than invited chat sessions, perhaps through the stage tailored groups to which all intervention participants were assigned. This would allow for those motivated to engage to do so and allow others to still view session material (eg, handouts, tip sheets, interactions between other participants and study counselors). Additionally, allowing participants to move between groups in response to an increasing stage of change was not used by many and potentially may undermine the social support generated from participating in a single group; thus, this strategy should likely not be used in the future.

Our feasibility study found that two-thirds of all Facebook intervention participants and one third of participants in the Precontemplation stage of change undertook one or more quit attempts, with each participant making at least one attempt reporting more than four on average. These findings are in line with results from daily assessments [53] and speak to the dynamic nature of the smoking cessation process and to the fact that many smokers need multiple quit attempts in order to attain smoking abstinence. However, although we used a standard definition of quit attempts (24 hours of purposeful abstinence), our inclusion of non-daily smokers could have led to overreporting of non-smoking days as quit attempts, although the assessment made clear that the definition included only purposeful attempts to quit smoking. Trying to quit without assistance other than the Tobacco Status Project intervention was much more popular than using a cessation aid such as medication among young adults. This is congruent with previous studies among young adults [54] and adolescents [55] and highlights the need to incorporate evidence-based treatment into media that are widely accessed by young people such as Facebook. Electronic cigarettes were used more frequently as a cessation aid than nicotine replacement therapy, even though clear evidence for their effectiveness is still lacking [56,57] and their use was not recommended in the intervention. Young adults may have strong expectations for the effectiveness of electronic cigarettes as a smoking cessation strategy [58]. Given the wide marketing of these products online to young adults [59], the high use in our sample is not surprising. Until convincing data on the effectiveness of electronic cigarettes for smoking cessation is presented, their use should not be recommended to young adults trying to quit smoking.

We tested a novel method of collecting biochemical verification of tobacco abstinence by mailing participants saliva cotinine test strips and asking them to send back pictures of test results. Incentives for assessment completion were not given until proof was sent. This strategy was successful for about half of those reporting abstinence at 3-, 6-, and 12-month assessments. Of the 13 cases for which we were unable to obtain biochemical verification of abstinence at any time point, 6 were unreachable, 1 had technical difficulties preventing transmission of verification data (photos), 1 had difficulty completing the test at home and results were seen as inconclusive, and 5 were not sent test kits due to errors in survey programming on the study end. This piloting of methods was valuable for informing quality control in our future efforts. Overall success with completing the biochemical procedure at home once test kits were sent was promising, and our strategy shows that validation measures are possible in an environment where information is increasingly easy to send as data (eg, through a smartphone). Notably, we did not have trouble recruiting young people into the study despite knowledge that the trial would ask for biochemical verification of abstinence, as has been a concern of others [60,61].

### Limitations

With a goal of determining feasibility and initial efficacy, this study was not adequately powered to fully test many of the relationships examined here. A larger, 2-group randomized trial powered to detect effects on primary outcomes and differences among incentive or motivation groups is warranted. Further, Facebook is a dynamic environment where design issues become out-of-date quickly. Potential changes to user agreements mean that researchers must be vigilant about privacy and confidentiality when working in this medium. Formative work with our target population indicated that young adult smokers were ashamed of smoking in many cases and wanted to keep the study involvement private from their larger Facebook social network [23]. This suggested the intervention should be conducted within the context of Facebook secret groups where only study administrators and group members have knowledge of their existence. Investigators then watched Facebook’s privacy practices to ensure that this did not change; if so, we would have considered a different context in which to run groups.

Another limitation is the general lack of sample diversity. In contrast to other online interventions in the United States or Canada that show a majority of female participants [62,63], our sample was primarily male. This was a surprise, given that a larger proportion of American women online use Facebook than men (77% vs 66%) [64]. There was no direct targeting of participants by gender in the Facebook advertising campaign, as one goal of this feasibility study was to determine the characteristics of participants who would enroll. Strategies are needed to recruit more female and ethnic minority participants. Facebook targeting can be used to place ads in locations including states and cities where more ethnic minority smokers reside. Advertising images can be used to target women and non-white smokers. Bull et al [65] recruited a large proportion of non-white participants into a Facebook sexual health intervention using respondent-driven sampling. Using a strategy to recruit “seed” participants and friends in their (real-world) social networks could generate samples that are diverse with respect to gender, ethnicity, and sexual orientation.

### Conclusions

Harnessing the popularity of social media to treat tobacco use in young adults holds great potential considering the overwhelming numbers using this medium daily. Focused on young adult smokers, a challenging group to engage, our study’s high retention and usability ratings suggest the Facebook quit smoking intervention is attractive and feasible to deliver. Early efficacy data are encouraging and support further investigation in a larger sample with a randomized design.

## Appendix

Multimedia Appendix 1 Altruism measure.

## Abbreviations

ANOVA: analysis of variance
CBT: cognitive behavioral therapy
ENDS: electronic nicotine delivery systems
FDA: Food and Drug Administration
HIPAA: Health Insurance Portability and Accountability Act
IQR: interquartile range
