# Supplementary material for: Feasibility and Quit Rates of the Tobacco Status Project: A Facebook Smoking Cessation Intervention for Young Adults
Source: J Med Internet Res. 2015 Dec 31;17(12):e291. doi: 10.2196/jmir.5209 (PMC4736286; doi:10.2196/jmir.5209)
Supplement: Multimedia Appendix 1 [file jmir_v17i12e291_app1.pdf]

# Appendix 1. Altruism measure

Imagine yourself in a situation in which you can keep or give to another person, all or any portion of $10. You may give money only in increments of $1. For example, you can give $0 and keep $10, or give $2 and keep $8, or give $8 and keep $2. These are only hypothetical examples, and the decision of how much to give is entirely yours.

Please imagine the situation to be as close as possible to a real-life situation.

Items

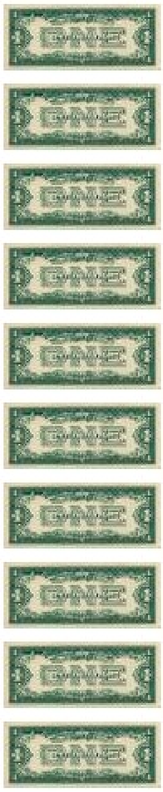

| You | Someone Else |
|---|---|
